# The microbiota diversity of *Festuca sinensis* seeds in Qinghai-Tibet Plateau and their relationship with environments

**DOI:** 10.3389/fmicb.2022.956489

**Published:** 2022-08-03

**Authors:** Yue Gao, Youjun Chen, Yang Luo, Junying Liu, Pei Tian, Zhibiao Nan, Qingping Zhou

**Affiliations:** ^1^State Key Laboratory of Grassland Agro-Ecosystems, Lanzhou University, Lanzhou, China; ^2^Key Laboratory of Grassland Livestock Industry Innovation, Ministry of Agriculture and Rural Affairs, Lanzhou University, Lanzhou, China; ^3^College of Pastoral Agriculture Science and Technology, Lanzhou University, Lanzhou, China; ^4^Institute of Qinghai-Tibetan Plateau, Southwest Minzu University, Chengdu, China

**Keywords:** *Festuca sinensis*, seed microbiota, environmental factors, temperature, precipitation, *Epichloë sinensis* infection rate

## Abstract

A total of 14 *Festuca sinensis* seed lots were collected from different geographical locations on the Qinghai-Tibet Plateau to study the seed microbiota and determine the abiotic (temperature, precipitation, and elevation) and biotic (*Epichloë sinensis* infection rate) factors likely to shape the seed microbiome. The 14 seed lots had different bacterial and fungal structures and significantly different diversities (*p* < 0.05). The α-diversity indices of the bacteria were significantly correlated with precipitation (*p* < 0.05), whereas those of the fungi were significantly correlated with temperature (*p* < 0.05). Microbiota analysis showed that Proteobacteria, Cyanobacteria, and Bacteroidetes were the most abundant bacteria at the phylum level in the seeds, and Ascomycota and Basidiomycota were the most abundant fungi. β-diversity analysis suggested large differences in the microbial communities of each sample. Redundancy analysis showed that temperature and precipitation were the main environmental factors that drive variations in the microbial community, at the medium-high elevation (3,000–4,500 m), the impact of temperature and precipitation on microbial community is different, and the other elevations that effect on microbial community were basically identical. Spearman's correlation analysis showed that the relative abundances of the most abundant bacterial phyla were significantly correlated with temperature (*p* < 0.05), whereas those of the most abundant fungal phyla were significantly correlated with precipitation (*p* < 0.05). *E. sinensis* infection rates were significantly correlated with elevation and temperature (*p* < 0.05). These results suggest that temperature and precipitation are the key factors driving the microbial community, that temperature and elevation also had a great influence on the *E. sinensis* infection rate, and that environmental factors (temperature and elevation) may further affect the microbial community by regulating the *E. sinensis* infection rate.

## Introduction

*Festuca sinensis*, a native cool-season perennial grass species, is distributed across cold and semi-arid regions of China. It is important for grassland production and establishment, restoration of degraded grasslands, and ecological management of the Qinghai-Tibet Plateau of China (Lin et al., 2018). Seeds represent one of the most crucial stages in a plant's life history (Finch-Savage and Bassel, 2015). Microorganisms inside and on the surface of seeds play the important roles in the germination and development of seedlings (Nelson, 2018). During seed germination, a new compartment of microbiome is created. Recent omics-based analyses have shown that plant seeds contain beneficial plant-genotype-specific microbes, which can be vertically transmitted from one plant generation to the next (Johnston-Monje et al., 2016; Adam et al., 2018). Therefore, seed microbes play the important roles in the seed itself and in plant growth (Hashsham et al., 2000). Although studies have reported the importance of seed microbiota, there are no reports on the microbiota of *F. sinensis* seed. Therefore, research on *F. sinensis* seeds and their microbiota is of great significance for the production and applications of *F. sinensis*.

Some studies have shown that microbial communities are significantly affected by plant species, the host environment, host genotype, host age, and many other factors (Tannenbaum et al., 2020). Environmental factors, such as temperature, precipitation, and elevation, could be the main factors that affect microbial diversity (Jiang et al., 2016). However, most reports have only focused on the relationships between environmental factors and the soil microbial community. Some studies have found that the soil microbial community may be affected by total carbon (C) and total nitrogen (N) in the soil (Schimel et al., 2007). Some studies have shown that different tillage practices for wheat (*Triticum aestivum*) can change the microbial diversity in the soil (Lupwayi et al., 1998). However, few studies have highlighted the effects of environmental factors on the microbial diversity of seeds. Klaedtke et al. (2016) found that environmental factors had an important influence on the structure of seed microbiota. However, the relationships between environmental factors (such as temperature, precipitation, and elevation) and microbial diversity of *F. sinensis* seeds have not been reported.

*F. sinensis* is frequently infected by an asexual, symptomless *Epichloë* species that has been identified as *Epichloë sinensis* (Song et al., 2015; Tian et al., 2020). The recent studies have produced conflicting results regarding *Epichloë* endophytes in pasture microbiomes. For example, Nissinen et al. (2019) demonstrated that resident *E. coenophiala*, as a keystone species, had divergent effects on bacterial and fungal communities in the leaf endosphere of *F. arundinacea* and shaped fungal but not bacterial communities. However, Tannenbaum et al. identified the effects of *E. festucae* var. *lolii* on bacterial microbiomes of pooled young perennial ryegrass seedlings (Tannenbaum et al., 2020). These conflicting results suggest that more research on the influence of *Epichloë* endophytes on microbial communities is needed to understand their mechanisms. Therefore, it is of substantial importance to study the microbiota diversity of different *F. sinensis* seeds and their relationships with *E. sinensis* infection rates to clarify the effects of *Epichloë* endophyte infection on the host seed microbiota.

As environmental factors are very important for the formation of seed microbiota, we wanted to clarify the effects of varied environmental conditions on *F. sinensis* seed microbiota, using seeds collected from different locations in the Qinghai-Tibet Plateau. Therefore, the aims of this study were to 1) identify the seed microbiome diversity of *F. sinensis* seeds, 2) identify the abiotic and biotic factors that are likely involved in shaping the microbiome of *F. sinensis* seeds, and 3) reveal the relationships among *E. sinensis* infection rate, the microbiota, and environmental factors.

## Materials and methods

### Seed materials

A total of 14 seed lots of *F. sinensis* were collected from different locations on the Qinghai-Tibet Plateau, as shown in Table 1 and Supplementary Figure S1. The *E. sinensis* infection rate in these seed lots was detected by the aniline blue staining method (Nan, 1996). A total of 100 seeds per seed lot were tested, and the *E. sinensis* infection rate was calculated (Christensen et al., 2008).

**Table 1:** Festuca sinensis seeds collected from different locations in Qinghai-Tibet Plateau.

**Ecotypes**	**Elevation/m**	***Epichloë sinensis* infection (%)**	**Longitude (E)**	**Latitude (N)**	**Location**
S1	2,589	64	101°57 ′234″	36 ° 21 ′641″	Sanhe Town, Ping'an Country Qinghai
S2	2,741	80	101 ° 57 ′437″	36 ° 20 ′'047″	Sanhe Town, Ping'an Country Qinghai
S3	2,912	72	97 ° 99 ′044″	37 ° 17 ′138″	Keyukezhen Town, Delingha City Qinghai
S4	2,912	48	97 ° 99 ′044″	37 ° 17 ′138″	Keyukezhen Town, Delingha City Qinghai
S5	2,994	80	102°06 ′348 ″	36 ° 20 ′508″	Bazanggou Township, Ping'an Country Qinghai
S6	3,060	56	101 ° 58 ′846″	36 ° 17 ′059″	Gucheng Township, Ping'an Country Qinghai
S7	3,129	73	102° 06 ′214 ″	36 ° 20 ′021″	Bazanggou Township, Ping'an Country Qinghai
S8	3,534	74	93 ° 07 ′325″	29 ° 58 ′065″	Gubo'gyamda Country, Nyingchi City,Tibet
S9	4,003	32	91 ° 54 ′782″	29 ° 44 ′692″	Zhaxigang, Maizhokunggar County, Tibet
S10	4,542	0	91 ° 53 ′580″	31 ° 20 ′039″	Luoma Town, Naqu City, Tibet
S11	4,617	52	93 ° 57 ′485″	35 ° 32 ′876″	Qumahe Township, Qumaleb country Qinghai
S12	4,617	20	93 ° 57 ′485″	35 ° 32 ′876″	Qumahe Township, Qumaleb country Qinghai
S13	4,897	54	91 ° 50 ′961″	29 ° 46 ′564″	Zaxigang, Maizhokunggar County, Tibet
S14	5,197	0	91 ° 55 ′336″	32 ° 54 ′246″	Tanggula Mountains, Golmud City, Qinghai

### DNA extraction

Each seed lot had three replicates, with 50 mg of seeds per replication. The total genomic DNA of each replication was extracted using the CTAB/SDS method after homogenization using a pestle and mortar (Ren et al., 2006). Seed powder (50 mg) was placed in a 2-ml microcentrifuge tube containing 1,000 μl of CTAB buffer and 20 μl of lysozyme. After the sample incubated at 65°C for 2–3 h with occasional mixing, the sample was vortexed briefly and then centrifuged for 10 min at 12,000 rpm. Then, 950 μl of the supernatant was mixed with 950 μl chloroform/isoamyl alcohol (24:25 v/v). Again, after totally mixing by vortexing, the sample was centrifuged for 10 min at 12,000 rpm. After repeating this step, the supernatant was mixed with ice-cold isopropanol (the volume of ice-cold isopropanol is three-quarters of the volume of the supernatant), and the tube was inverted five times to precipitate nucleic acids. The sample was centrifuged at 12,000 rpm for 10 min, and the precipitate was washed two times with 1 ml of 75% ethanol. The remaining small amount of liquid was collected by further centrifugation and then sucked out with a pipette tip. The pellets were air-dried for 2 h. Then, 50 μl ddH_2_O was added to dissolve DNA samples. Then, 1 μl RNase A was added to digest RNA, and the mixture was incubated at 37°C for 15 min. DNA was diluted to 1 μg/L with sterile water after its purity was quantified using a NanoDrop 2000 spectrophotometer (Thermo Fisher Scientific, Waltham, MA, USA) (Zheng et al., 2018).

### Polymerase chain reaction amplification and high-throughput sequencing

Specific primers with barcodes were synthesized to amplify the bacterial 16s rRNA V4–V5 region (515F:5'-ACTCCTACGGGAGCAGCA-3'; 806R:5'-GGACTACHVGGGTWTCTAAT-3') and the fungal ITS1 or ITS2 region (ITS5-1737F: 5'-CTTGGTCATTTAGAGGAAGTAA-3'; ITS2-2043R: 5'-GCTGCGTTCTTCATCGATGC-3') to assess the composition of both bacterial and fungal communities (Klindworth et al., 2013). Phusion^®^ Hi-Fi PCR Master Mix (New England Biolabs, Ipswich, MA, USA) was used for all PCR. Each PCR mixture contained 5 μl genomic DNA (40–60 ng), 1.5 μl forward primer (10 μm), 1.5 μl reverse primer (10 μM), 1 μl Toyobo, 1 μl KOD FX Neo Buffer (2X), 10 μl dNTP (2 mM), and ddH_2_O was added for a total volume of 50 μl. PCR was performed under the following conditions: one cycle at 95°C for 10 min, 15 cycles at 95°C for 1 min, 50°C for 1 min, 72°C for 1 min, and finally 72°C for 7 min. PCR products were subjected to 2% agarose gel electrophoresis for purification and magnetic beads were purified using a TruSeq^®^DNA PCR-Free Sample Preparation Kit (Illumina, San Diego, CA, USA). A DNA library was constructed after homogenization and quantified using Qubit and q-PCR (AB 9902). After the library was qualified, NovaSeq6000 was used for sequencing at Genepioneer Biotechnologies (Nanjing, China) with separate runs for the 16S rRNA and ITS amplicon pools (Reuter et al., 2015). All quality sequence files supporting the findings of this study are available in the NCBI Sequence Read Archive (SRA) under BioProject ID PRJNA844205.

### Microbial diversity analysis

To maximize the quantity and quality of reliable sequences, the following protocols were performed. TrimGalore (version 0.4.2) software filtered out bases with a terminal mass of less than 20, which might comprise adapter and short sequences less than 100 bp in length. After merging *via* FLASH2 (V1.2.7) software and removing low-quality sequences, the primer sequences were trimmed using Mothur (version 1.41.1) (Schloss et al., 2009). Sequences less than 100 bp in length or with an error rate of more than two were discarded by USEARCH (version 10.0). High-quantity and high-reliability sequences were clustered into operational taxonomic units (OTUs) with 97% similarity using the UPARSE algorithm and singleton OTUs (with only one read) were removed.

Bacterial and fungal OTU representative sequences were classified taxonomically through BLAST alignment against the SSUrRNA database of SILVA138 (http://www.arb-silva.de/) and the Unit (v8.2) fungal ITS database (http://unite.ut.ee/), respectively (Bryant and Frigaard, 2006; Liu et al., 2012). The OTU tables were rarefied to the sample containing the lowest number of sequences, with a threshold of >10,000 sequences (all samples with < 10,000 sequences were removed from analyses prior to the rarefaction step). A subsequent analysis of diversity was performed based on the output-normalized data. QIIME software (version 1.9.1) was used to compute the diversity indices of Chao1, Simpson, Shannon, and the abundance-based coverage estimator (ACE) based on OTU numbers to study the diversity and structure of the microbial community of *F. sinensis* seeds (Shade et al., 2017).

Unweighted pair-group method with arithmetic means (UPGMA) cluster analysis was performed to interpret the distance matrix using average linkage which conducted using the QIIME software (version 1.9.1). Principal component analysis (PCA) based on the unweighted UniFrac distances (Bray–Curtis) (QIIME software, version 1.9.1) was used to examine the differences in microbial community structures among 14 seed lot samples based on the relative abundances of OTUs. PCA was performed using the Ade4 package and ggplot2 package of R software (Version 2.15.3).

### Statistical analysis

Meteorological data were obtained from the China Meteorological Center (http://data.cma.cn). Based on the longitude, latitude, and elevation of each sampling point, the thin plate smoothing spline algorithm in package Anusplin 4.4 (Version 4.4, Canberra, Australia http://fennerschool.anu.edu.au/files/anusplin44.pdf) was used to calculate the kriging difference to obtain the monthly mean temperature (MMT) and monthly mean precipitation (MMP) at each sampling point from 2006 to 2015. The growing season of *F. sinensis* is from April to August, and the average temperature and precipitation from April to August were calculated as the mean temperature and precipitation during the growing season. Microsoft Excel 2020 was used to calculate the MMT, MMP, growing monthly mean temperature (GMMT), and growing monthly mean precipitation (GMMP). Spearman's correlation analysis in SPSS 23.0 (version 23.0; SPSS Inc, Chicago, IL, USA) was used to analyze the relationship among environmental factors, *E. sinensis* infection rate, and microbial diversity, and statistical significance was set at *p* < 0.05.

One-way analysis of variance (ANOVA) was used to determine statistically significant differences in microbial community diversity among the 14 groups using SPSS 23.0 (version 23.0; SPSS Inc, Chicago, IL, USA). When ANOVA indicated a significant difference, Fisher's least significant difference (LSD) test was applied to conduct multiple pairwise comparisons, and statistical significance was set at *p* < 0.05.

Redundancy analysis (RDA) was performed using Canoco 5 software (Microcomputer Power, Ithaca, New York, USA), as discussed by Braak (1994). In RDA, microbial parameters were used as “species,” and the ordination axes were constrained to be linear combinations of the environmental factors (i.e., MMT, MMP, GMMT, and GMMP). Thus, this analysis allowed the relationships between environmental factors and microbial parameters to be directly compared. Using the Monte Carlo permutation test (number of permutations), the significance of the environmental factors in accounting for the observed variance in the microbial parameters can be assessed with *p*-values. In the RDA diagram, positively correlated variables are indicated by arrows pointing in the same direction, negatively correlated variables point in opposite directions, and perpendicular variables are uncorrelated. In addition, the arrow length is a measure of the relative importance of environmental factors in explaining the variances of the microbial parameters. To more clearly understand the impacts of elevation, the samples were divided into 3 groups, including high elevation (2,500–3,000 m), medium-high elevation (3,000–4,500 m), and extremely high elevation (4,500–5,500 m). Additionally, a total of 14 samples were also analyzed together and compared.

## Results

### Sequencing annotation

After 16S rRNA sequencing, an average of 104,769 tags was detected for each sample, and 65,201 tags were obtained on average for each sample after quality control, with the efficiency of quality control reaching 62.28%. Sequences were clustered into OTUs with 97% identity, and a total of 3,003 OTUs were obtained. Taxonomic annotations of these OTUs were conducted using the SILVA138 database. There were 2,862 (95.30%) OTUs that could be annotated in the database.

After ITS sequencing, an average of 103,696 tags was detected for each sample. The quantity of effective data under quality control reached 65,156 for each sample, and the quality control efficiency reached 62.88%. The OTUs were clustered with 97% identity and 636 OTUs were identified. Taxonomic annotation was performed using the OTUs sequence and the UNITE databases, and 592 (93.08%) OTUs were annotated.

### Microbiota diversity in seeds

#### OTUs level

Both the rarefaction of the sequencing depth (Supplementary Figure S2) and coverage estimators (Supplementary Table S2) indicated that the sequencing depth was sufficient to represent the microbial composition of the samples and to reveal the microbial community structure.

**Figure 1:**
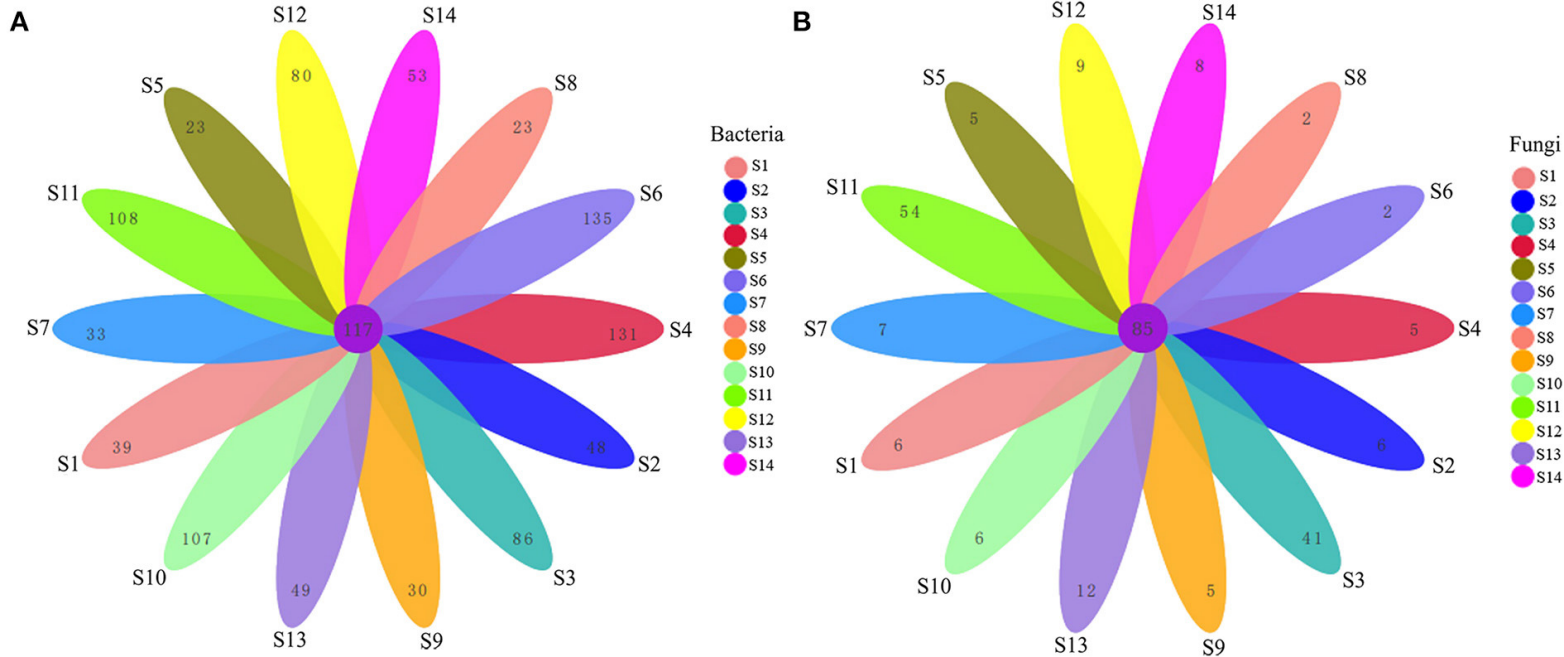
Venn petals of bacteria **(A)** and fungi **(B)** at the OTUS level showing the number of commonly and uniquely expressed genes collected the *Festuca sinensis* seeds from different geographical locations.

A total of 2,862 bacterial OTUs were obtained by clustering, whereas a total of 1,062 bacterial OTUs were obtained by clustering in the Venn petals (Figure 1A). In addition, 117 OTUs were shared by bacterial communities of these 14 seed lots, accounting for 11.02% of the total number of OTUs. Ecotype S6 had the largest number of unique OTUs (252), followed by ecotypes S4 (248), and S11 (225). Ecotypes S5 and S8 had the lowest numbers of unique OTUs (170), followed by ecotype S7 (180). A total of 592 fungal OTUs were obtained by clustering, whereas a total of 253 fungal OTUs were obtained by clustering in the Venn petals (Figure 1B), and 85 OTUs were shared by fungal communities of these 14 seed lots, accounting for 33.60% of the total number of OTUs. Ecotype S11 had the highest number of unique OTUs (139), followed by ecotype S3 (126). Ecotypes S6 (87), S8 (87), S9 (90), and S4 (90) had the lowest numbers of OTUs.

#### Relative abundance of the bacterial and fungal phyla

**Figure 2:**
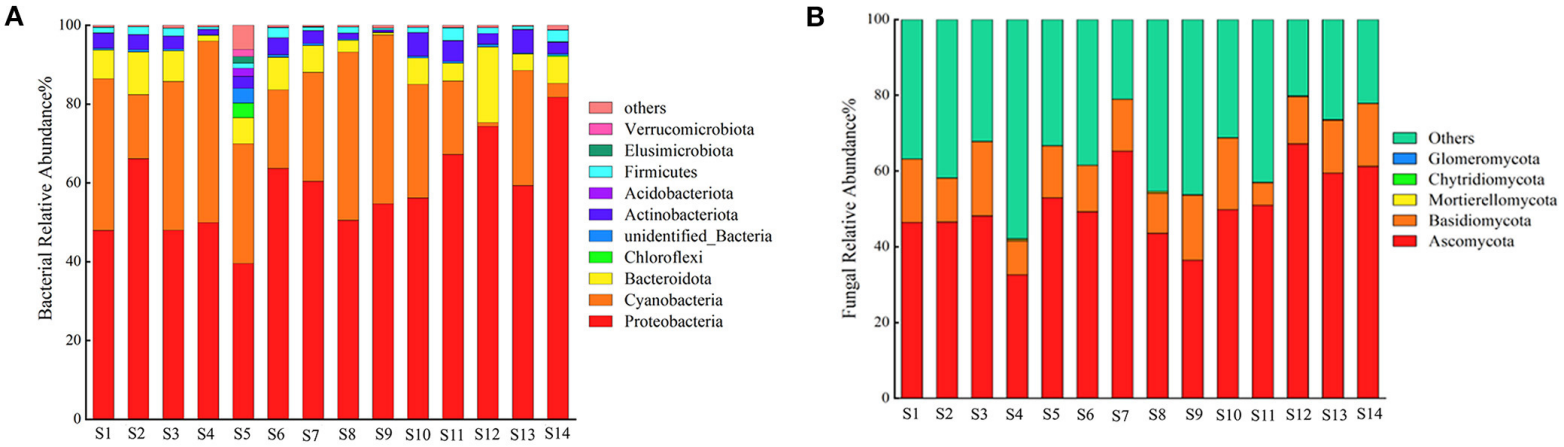
The relative abundance of the top 10 phyla of bacteria in seed lot from 14 ecotypes 16s ribosomal RNA sequencing **(A)** and fungi in seed lot from 14 ecotypes ITS sequencing **(B)**.

The relative abundances of the bacterial phyla are shown in Figure 2A. These phyla included Proteobacteria, Cyanobacteria, Bacteroidetes, Chloroflexi, Actinobacteria, Acidobacteria, Firmicutes, Elusimicrobiota, and Verrucomicrobiota. Proteobacteria, Cyanobacteria, and Bacteroidetes were the most abundant phyla. The microbiomes of each seed lot were different. The relative abundances of Proteobacteria in seeds of ecotypes S2, S6, S7, S11, S12, S14, S8, S9, S10, and S13 were over 50%, and Proteobacteria was the most abundant bacterial phylum in the seeds. The relative abundances of the three most abundant phyla in each seed lot are shown in Supplementary Table S2. The relative abundances of Proteobacteria in the seeds of ecotype S13 were significantly higher than those in the seeds of ecotypes S1, S4, S3, S5, S7, S11, S12, S14, and S10 (*p* < 0.05). The relative abundance of Cyanobacteria in the seeds of ecotype S3 was significantly higher than those in the seeds of ecotypes S2, S9, and S13 (*p* < 0.05). The relative abundance of Bacteroidota in seeds of ecotype S9 was highest and was significantly higher than those in seeds of the other ecotypes (*p* < 0.05). The relative abundance of Chloroflexi in the seeds of ecotype S5 was significantly higher than those in the seeds of the other ecotypes (*p* < 0.05).

The relative abundances of the fungal phyla are shown in Figure 2B. These phyla include Ascomycota, Basidiomycota, Mortierellomycota, Chytridiomycota, and Glomeromycota. Ascomycota and Basidiomycota were the most abundant fungi, with relative abundances of > 50%. Ecotype S9 had the highest relative abundance of Ascomycota (Supplementary Table S2), which was significantly higher than those in seeds of ecotypes S3 and S12 (*p* < 0.05). The relative abundance of Ascomycota in seeds of ecotype S3 was significantly lower than those in seeds of ecotypes S7 and S9 (*p* < 0.05). The relative abundances of Basidiomycota in seeds of ecotypes S4 and S14 were significantly higher than those in seeds of ecotypes S3 and S8 (*p* < 0.05), and the relative abundance of Basidiomycota in seeds of ecotype S8 was significantly lower than that in seeds of ecotypes S1, S4, S12, and S14 (*p* < 0.05).

#### α-diversity of bacterial and fungal communities in seeds

There were significant differences among α-diversity indices of the bacteria of different ecotypes (Table 2). The Chao richness index of ecotype S116 was significantly higher than those of ecotypes S2, S12, S8, S9, and S13 (*p* < 0.05). The Shannon index of ecotype S9 was significantly higher than that of ecotype S14 (*p* < 0.05). The Simpson indices of ecotypes S2, S5, S6, S14, S8, and S9 were significantly higher than those of ecotypes S3 and S12 (*p* < 0.05). The ACE indices of ecotypes S4, S3, and S11 were significantly higher than those of ecotypes S2, S5, S12, S8, S9, S10, and S13 (*p* < 0.05).

**Table 2:** α-diversity of bacterial and fungal communities in seeds.

**Ecotypes**	**Bacteria**				**Fungi**			
	**Chao1**	**Shannon**	**Simpson**	**ACE**	**Chao1**	**Shannon**	**Simpson**	**ACE**
S1	505.6 ± 23.1 abcd	3.9 ± 0.8ab	0.8 ± 0.1abcd	513.6 ± 22.7abc	158.0 ± 3.9ab	3.7 ± 0.1ab	0.9 ± 0.0a	160.1 ± 4.1ab
S2	454.2 ± 31.0bcd	4.4 ± 0.6ab	0.8 ± 0.1ab	459.1 ± 31.7bc	161.0 ± 3.9ab	3.6 ± 0.1ab	0.9 ± 0.0a	163.3 ± 3.4ab
S3	571.9 ± 70.0abcd	2.8 ± 0.1ab	0.7 ± 0.0cd	589.3 ± 72.8ab	181.2 ± 18.9ab	3.3 ± 0.0abc	0.8 ± 0.0ab	179.6 ± 16.8ab
S4	680.8 ± 96.1a	4.1 ± 0.2ab	0.8 ± 0.0abc	692.5 ± 96.1a	159.0 ± 7.5ab	3.4 ± 0.1abc	0.8 ± 0.0ab	164.0 ± 8.1ab
S5	489.3 ± 21.3abcd	4.6 ± 1.1ab	0.8 ± 0.1ab	499.3 ± 19.4bc	172.3 ± 10.0ab	3.8 ± 0.1a	0.9 ± 0.0a	171.3 ± 8.7ab
S6	655.1 ± 112.6ab	4.3 ± 0.6ab	0.8 ± 0.1ab	581.8 ± 53.8abc	156.0 ± 9.6ab	3.5 ± 0.2abc	0.9 ± 0.0ab	158.9 ± 9.8ab
S7	529.8 ± 20.8abcd	4.0 ± 0.2ab	0.8 ± 0.0abc	542.5 ± 21.0abc	154.0 ± 10.4ab	3.4 ± 0.2abc	0.8 ± 0.0ab	157.0 ± 11.2ab
S8	389.6 ± 61.2d	4.0 ± 0.2ab	0.8 ± 0.0ab	392.5 ± 64.8c	129.7 ± 7.1b	3.1 ± 0.08bc	0.8 ± 0.01ab	126.3 ± 5.0b
S9	416.5 ± 49.5cd	5.1 ± 0.5a	0.9 ± 0.0a	422.7 ± 48.2bc	156.9 ± 5.4ab	3.5 ± 0.21abc	0.8 ± 0.03ab	160.5 ± 5.5ab
S10	471.8 ± 105.4abcd	3.6 ± 0.2ab	0.8 ± 0.0abcd	478.9 ± 109.1bc	176.5 ± 10.9ab	3.6 ± 0.20abc	0.8 ± 0.03ab	179.8 ± 10.6ab
S11	604.2 ± 55.5abc	3.3 ± 0.1ab	0.7 ± 0.01bcd	614.8 ± 56.9ab	205.7 ± 7.2a	3.4 ± 0.11abc	0.8 ± 0.02ab	210.7 ± 7.9a
S12	421.2 ± 41.2cd	2.5 ± 0.1ab	0.7 ± 0.0d	431.1 ± 38.9bc	178.5 ± 22.6ab	3.4 ± 0.20abc	0.8 ± 0.02ab	181.7 ± 23.1ab
S13	430.7 ± 55.1cd	4.2 ± 0.4ab	0.8 ± 0.0abc	435.5 ± 53.7bc	158.8 ± 28.2ab	2.9 ± 0.42c	0.7 ± 0.11b	161.1 ± 29.7ab
S14	551.2 ± 10.1abcd	4.1 ± 0.2b	0.8 ± 0.0ab	563.4 ± 9.3abc	185.7 ± 8.3ab	3.8 ± 0.06a	0.9 ± 0.01a	185.4 ± 5.5ab

The different lowercase letters in a column represent significant differences among treatments one-way ANOVA (p < 0.05).

The Chao richness index of fungi in ecotype S11 was significantly higher than that in ecotype S8 (*p* < 0.05; Table 2) and was not significantly different from that of other ecotypes. The Shannon indices of ecotypes S5 and S14 were significantly higher than those of ecotypes S8 and S13 (*p* < 0.05) and were not significantly different from those of the other ecotypes. Simpson indices of ecotypes S1, S2, S5, and S14 were significantly higher than those of ecotype S13 (*p* < 0.05) and were not significantly different from those of the other ecotypes. The ACE index of ecotype S11 was significantly higher than that of ecotype S8 (*p* < 0.05) and was not significantly different from those of the other ecotypes.

#### β-diversity of bacterial and fungal communities in seeds

**Figure 3:**
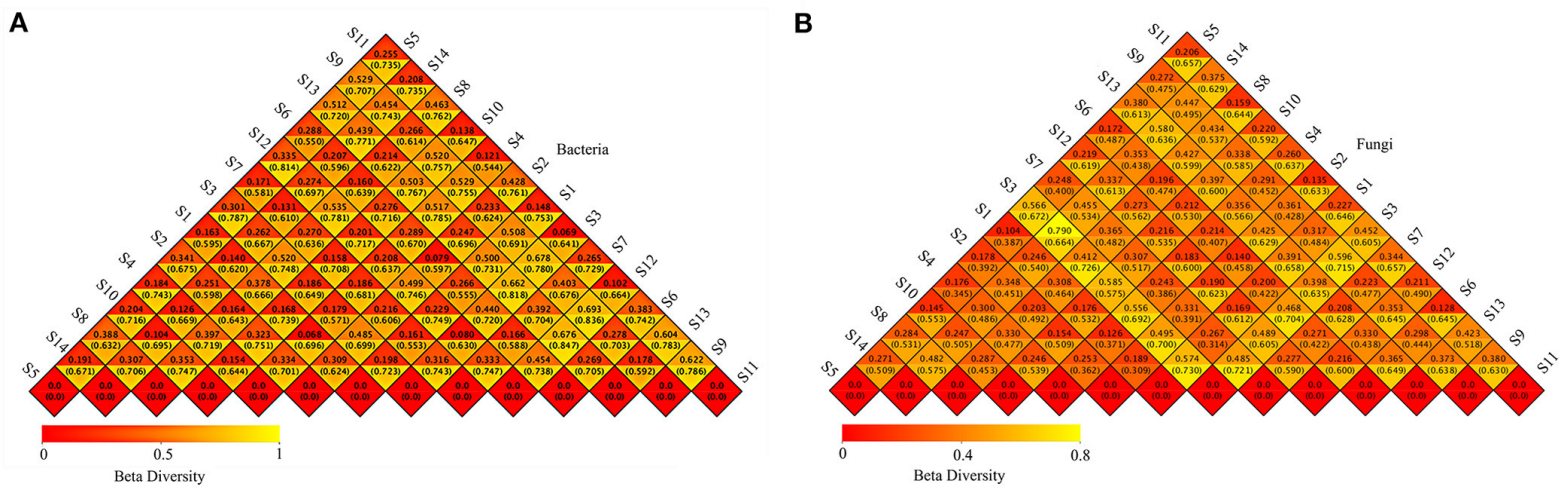
Beta diversity of bacterial **(A)** and fungal **(B)** heat map based on the UniFrac distance. The upper triangle is the weighted distance. The distance from the lower triangles is the unweighted distance.

A heat map of the beta diversity measurements is shown in Figure 3. For the bacterial community (Figure 3A), the distances of the samples based on weighted UniFrac were between 0.068 and 0.693, and the distances of the samples based on unweighted UniFrac were between 0.544 and 0.847. For the fungal community (Figure 3B), the distances of the samples based on weighted UniFrac were between 0.104 and 0.790, and the distances of the samples based on unweighted UniFrac were between 0.309 and 0.730. Overall, the UniFrac distances were relatively large.

#### UPGMA cluster and PCA

**Figure 4:**
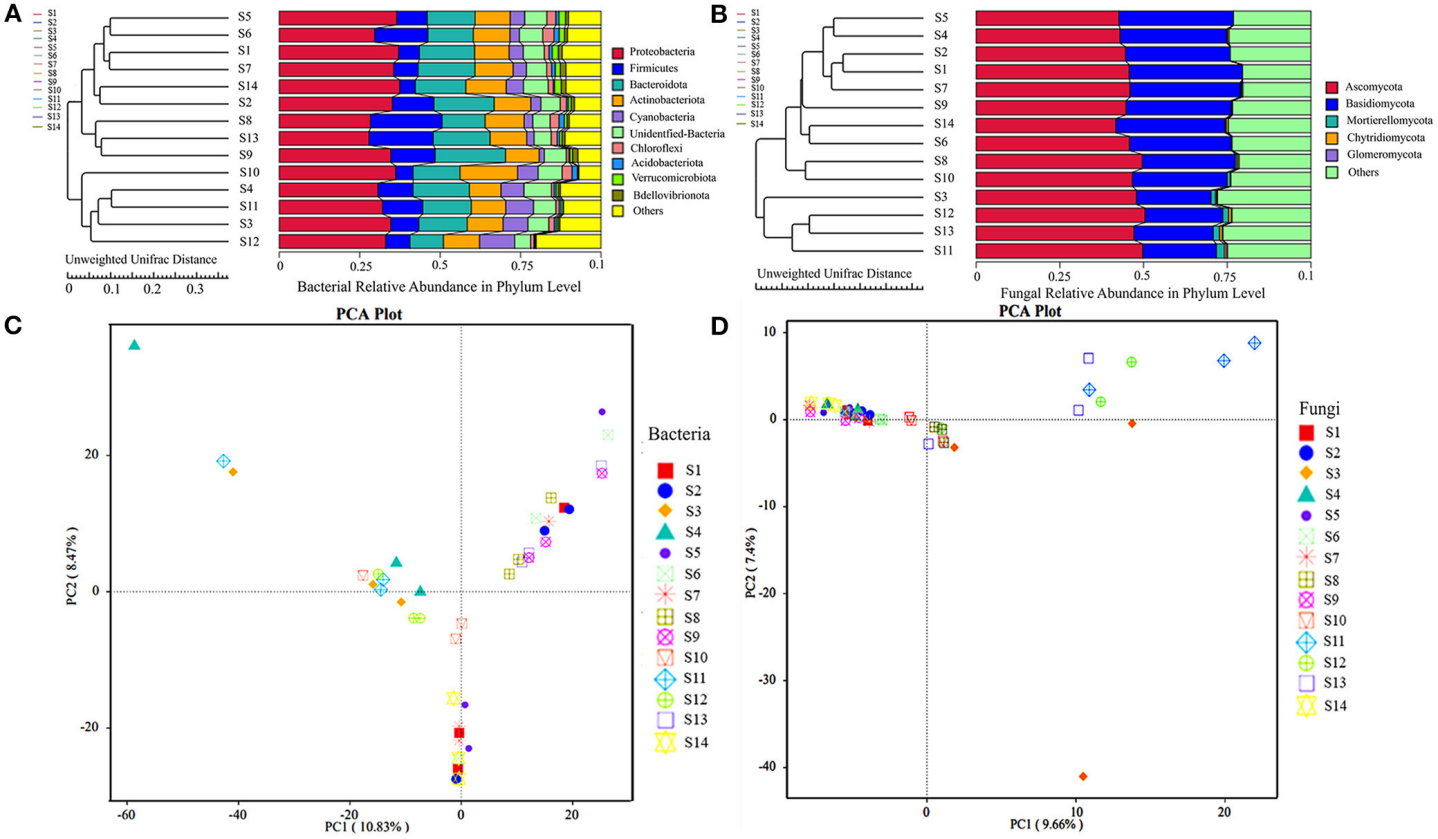
Analysis of *F. sinensis* seeds microbial communities with unweighted UniFrac. Principal component analysis (PCA) of bacterial **(A)** and fungal community **(B)** with unweighted UniFrac. Symbols represent different *F. sinensis* seeds. UPGMA tree based on unweighted UniFrac distance at the phylum level. Explanation: On the left is the UPGMA cluster tree structure, and on the right is the species relative abundance distribution at the phylum level for each sample. The phylogenetic relationship of the bacteria and fungi was determined by PCR sequencing portions of the 16S rRNA gene **(C)** and ITS gene **(D)**.

This study used both UPGMA cluster tree and PCA to cluster the *F. sinensis* seeds based on unweighted UniFrac (Figure 4). The UPGMA cluster analysis was performed using the unweighted UniFrac distance matrix, and the clustering results were integrated with the relative abundance column chart at the phylum taxon level for each sample. The phylogenetic tree showed that the bacterial communities were divided into four three groups (Figure 4A), and the fungal communities were divided into two major groups (Figure 4B), with each group containing samples from different areas of the Qinghai-Tibet Plateau. The KMO values for PCA of the bacteria (0.847, Supplementary Table S3) and fungi (0.938, Supplementary Table S3) are big enough to run multivariate analysis. For bacterial communities (Figure 4C), the first and second components explained 10.83 and 8.47% of the variances, respectively. For fungal communities (Figure 4D), the first and second components explained 9.66 and 7.4% of the variances, respectively. The results showed that the bacterial communities clustered more distinctively than the fungal communities. The bacterial communities of seed ecotypes S5, S1, S14, and S7 were less similar to those in seed ecotypes S8, S9, S2, S13, and S4, S11, S3, and S12, respectively. The fungal communities of seed ecotypes S11, S13, S12, and S3 were clustered together; however, they were less similar to those in seed ecotypes S10, S8, S6, S14, S9, S7, S1, S2, S4, and S5.

#### Correlation analysis

##### Correlation analysis between α-diversity and environmental factors

Analysis of correlations among α-diversity and environmental factors (Table 3) showed that the Chao richness index and ACE index of bacteria were significantly negatively correlated with MMP and GMMP (*p* < 0.05). However, the Simpson index of bacteria was significantly positively correlated with MMP and GMMP (*p* < 0.05). The Chao richness index and the ACE index of fungi were significantly negatively correlated with MMT and GMMT (*p* < 0.05). The *E. sinensis* infection rate was significantly positively correlated with MMT and GMMT (*p* < 0.05) and significantly negatively correlated with elevation (*p* < 0.05).

**Table 3:** Correlation coefficient between seed microbial α-diversity and environmental factors.

**Environmental factors**	**Bacteria**				**Fungi**				***Epichloë sinensis*** **infection rate**
	**Chao1**	**shannon**	**simpson**	**ACE**	**Chao1**	**shannon**	**simpson**	**ACE**	
MMT	−0.254	0.479	0.216	−0.293	−0.624^*^	0.251	0.280	−0.675^**^	0.652^*^
MMP	−0.620^*^	0.483	0.565^*^	−0.642^*^	−0.461	0.128	0.108	−0.437	0.022
GMMT	−0.165	0.470	0.212	−0.223	−0.642^*^	0.278	0.324	−0.697^**^	0.674^**^
GMMP	−0.620^*^	0.483	0.565^*^	−0.642^*^	−0.461	0.128	0.108	−0.437	0.022
Elevation	−0.244	−0.117	−0.004	−0.231	0.302	−0.238	−0.280	0.407	−0.636^*^
*Epichloë sinensis* infection rate	−0.077	0.262	0.131	−0.077	−0.399	−0.081	0.042	−0.526	x

“*” indicates a significant correlation at p < 0.05;

“**” indicates a very significant correlation at p < 0.01.

##### Correlation analysis of the relative abundances of the most abundant bacteria and fungi with environmental factors

The correlation of the relative abundances of the three most abundant bacterial phyla (Proteobacteria, Cyanobacteria, and Bacteroidetes) with environmental factors was analyzed (Table 4). The relative abundance of Proteobacteria was significantly positively correlated with MMP and GMMP (*p* < 0.05). The relative abundance of Cyanobacteria was very significantly negatively correlated with MMP and GMMP (*p* < 0.01). The relative abundance of Bacteroidetes was significantly positively correlated with MMT and GMMT (*p* < 0.05). The correlation of relative abundances of the two most abundant fungal phyla (Ascomycota and Basidiomycota) with environmental factors was also analyzed (Table 4). The relative abundance of Ascomycota was significantly positively correlated with MMP and GMMP (*p* < 0.05).

**Table 4:** Correlation coefficient between the relative abundance of the most abundance bacteria and fungi and environmental factors.

**Environmental factors**	**Bacterial abundance**			**Fungal abundance**	
	**Proteobacteria**	**Cyanobacteria**	**Bacteroidota**	**Ascomyota**	**Basidiomycota**
MMT	0.075	−0.395	0.534^*^	0.324	−0.018
MMP	0.547^*^	−0.712^**^	0.291	0.650^*^	0.088
GMMT	0.044	−0.364	0.542^*^	0.271	−0.049
GMMP	0.547^*^	−0.712^**^	0.291	0.650^*^	0.088
*Epichloë sinensis* infection rate	−0.040	−0.137	0.170	−0.128	−0.520
Elevation	0.390	−0.163	−0.383	0.385	0.143

Within a confidence interval of p = 0.05,

“*” indicates a significant correlation, and

“**” indicates a very significant correlation.

##### The relationship between the composition of microbial communities and environmental factors

Through Monte Carlo permutation, the ranking results of the four axes could better reflect the relationship between environmental factors and microbial communities (Supplementary Tables S4–S7). The relationships between environmental factors and abundances of bacterial and fungal at the phylum and genus levels were analyzed on the overall elevation, high elevation, medium-high elevation, and extremely high elevation (Supplementary Table S8). On the overall elevation, MMP and GMMT had significant effects on the variation in the bacterial phyla and genera (*p* < 0.05), with explained degrees of variation being 42.4, 21.0, 48.4, and 17.9%, respectively (Supplementary Table S8). MMP and MMT had a significant influence on the variation in the fungal phyla (*p* < 0.05, Supplementary Table S8), and their explained degrees of variation were 29.3 and 23.2%, respectively. MMP and GMMP had significant effects on the variation in the fungal genera, with explained degrees of variation of 21.7 and 17.8%, respectively (Supplementary Table S8). These results indicated that temperature and precipitation were the main environmental factors that affected the spatial differentiation of microbial communities. *E. sinensis* infection rates had a certain effect on the relative abundance of the phyla and genera of fungi (the explained degrees of variation were 7.4 and 9.2%, respectively). Elevation had the smallest effect on the phyla and genera of bacteria, with explained degrees of variation of only 1.0 and 0.4%, respectively. GMMT had the smallest effect on the phyla and genera of fungi, with explained degrees of variation of only 2.5 and 2.1%, respectively. On the high elevation, medium-high elevation and extremely high elevation the result of all axes were *F* < 0.1, *p* = 1 (Supplementary Table S8).

**Figure 5:**
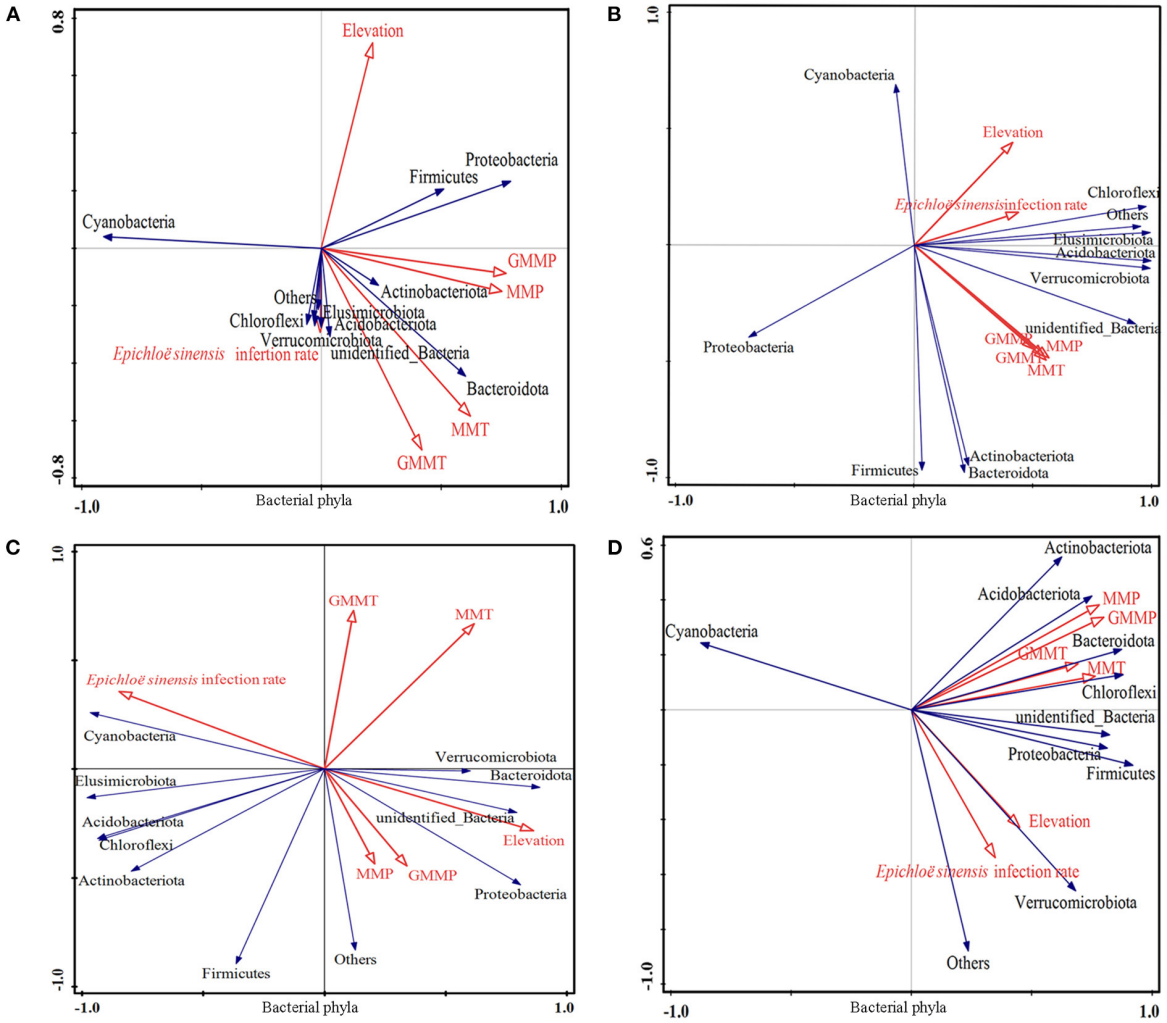
Effect of environmental factors on bacterial phyla. **(A)** on overall elevation (2,589–5,197 m). **(B)** on high elevation (2,589–3,000 m); **(C)** on medium-high elevation (3,000–4,500 m); **(D)** and on extremely high elevation (4,500–5,197 m). The microbial parameters (expressed as response variables in the RDA) were presented as black line vectors, and the environmental factors (explanatory variables) were presented as red line vectors. MMT, monthly mean temperature; MMP, monthly mean precipitation; GMMT, growing monthly mean temperature; GMMP, growing monthly mean precipitation.

**Figure 6:**
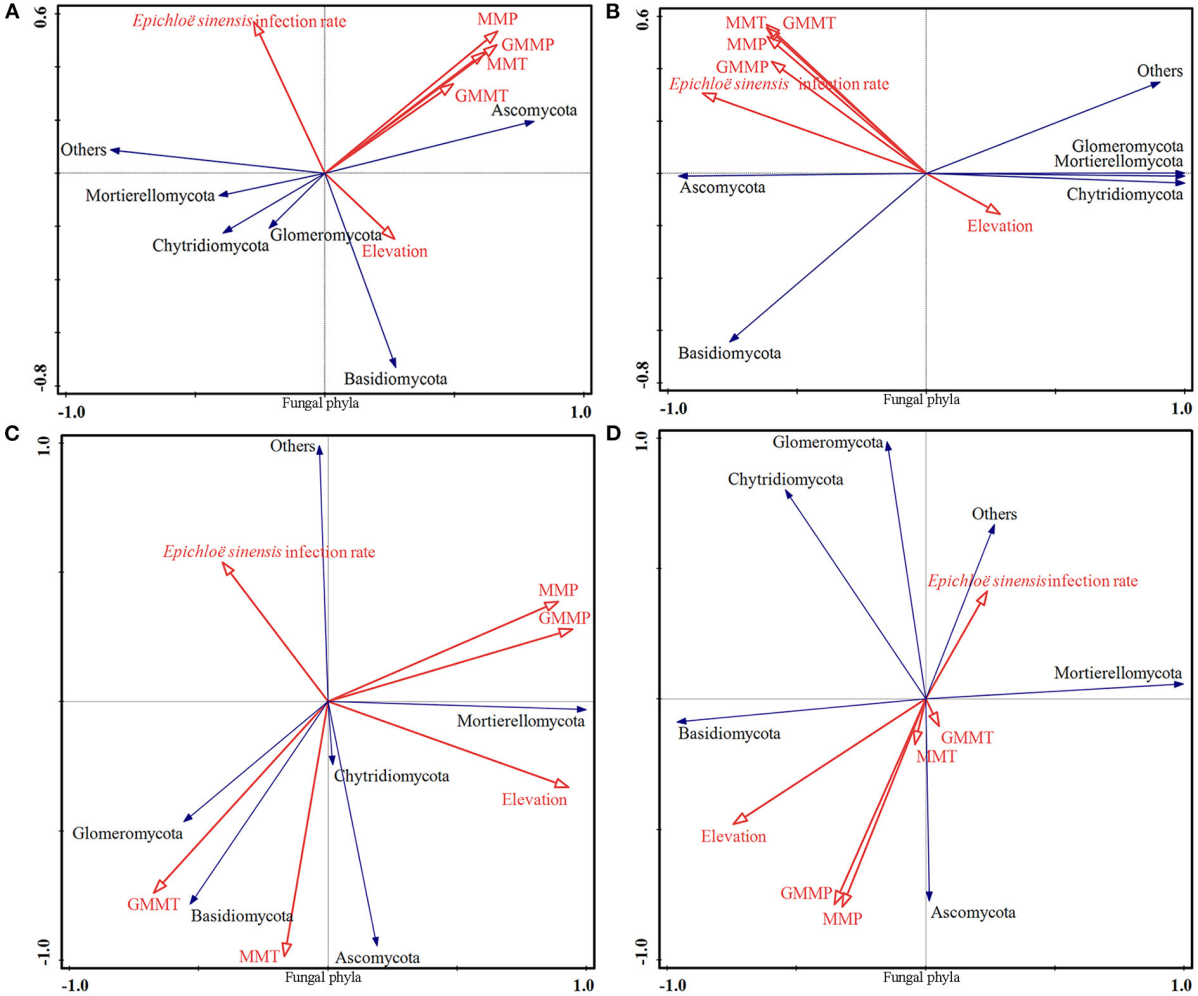
Effect of environmental factors on fungal phyla. **(A)** on overall elevation (2,589–5,197 m). **(B)** on high elevation (2,589–3,000 m); **(C)** on medium-high elevation (3,000–4,500 m); **(D)** and on extremely high elevation (4,500–5,197 m). The microbial parameters (expressed as response variables in the RDA) were presented as black line vectors, and the environmental factors (explanatory variables) were presented as red line vectors. MMT, monthly mean temperature; MMP, monthly mean precipitation; GMMT, growing monthly mean temperature; GMMP, growing monthly mean precipitation.

**Figure 7:**
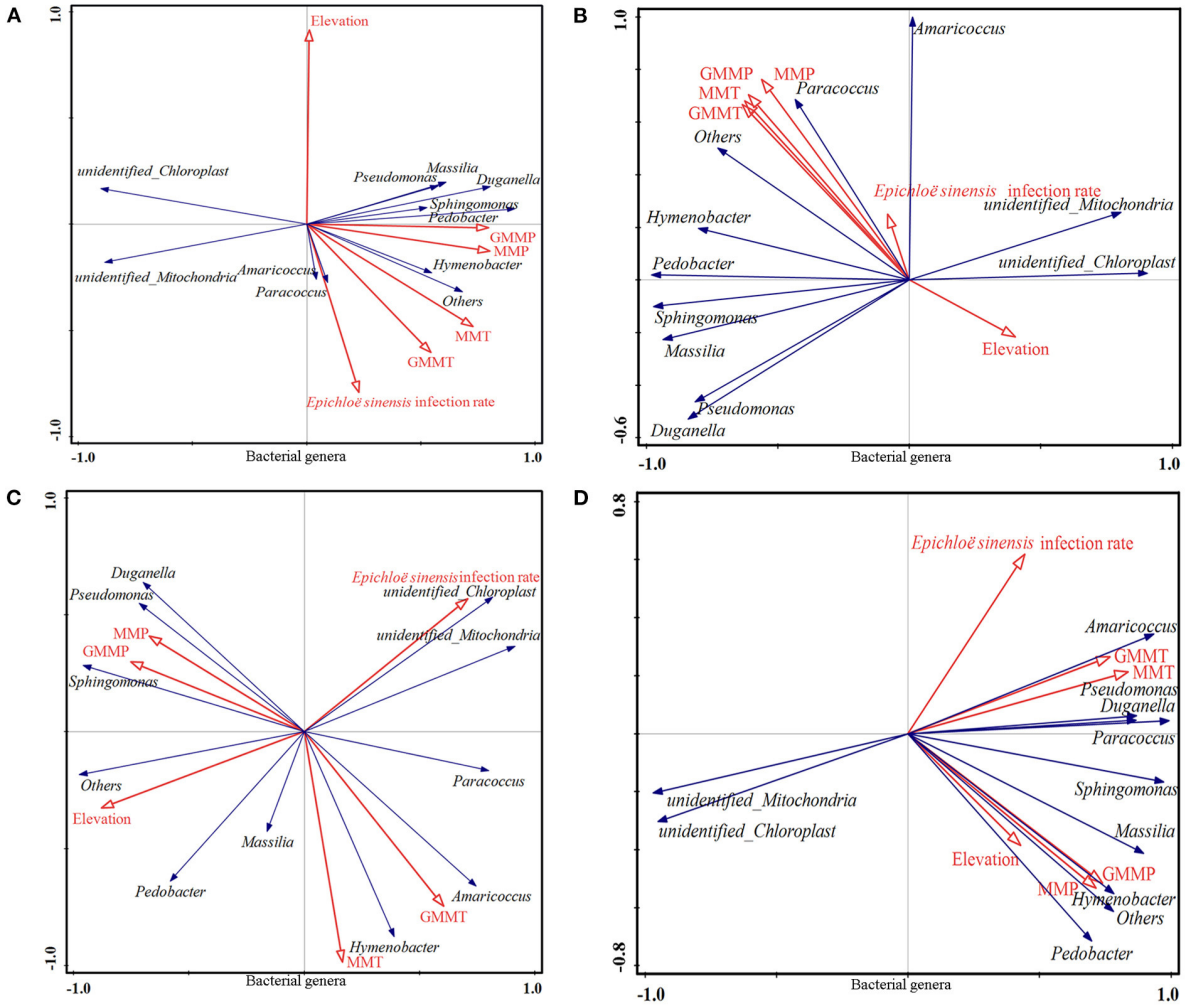
Effect of environmental factors on bacterial genera. **(A)** on overall elevation (2,589–5,197 m). **(B)** on high elevation (2,589–3,000 m); **(C)** on medium-high elevation (3,000–4,500 m); **(D)** and on extremely high elevation (4,500–5,197 m). The microbial parameters (expressed as response variables in the RDA) were presented as black line vectors, and the environmental factors (explanatory variables) were presented as red line vectors. MMT, monthly mean temperature; MMP, monthly mean precipitation; GMMT, growing monthly mean temperature; GMMP, growing monthly mean precipitation.

**Figure 8:**
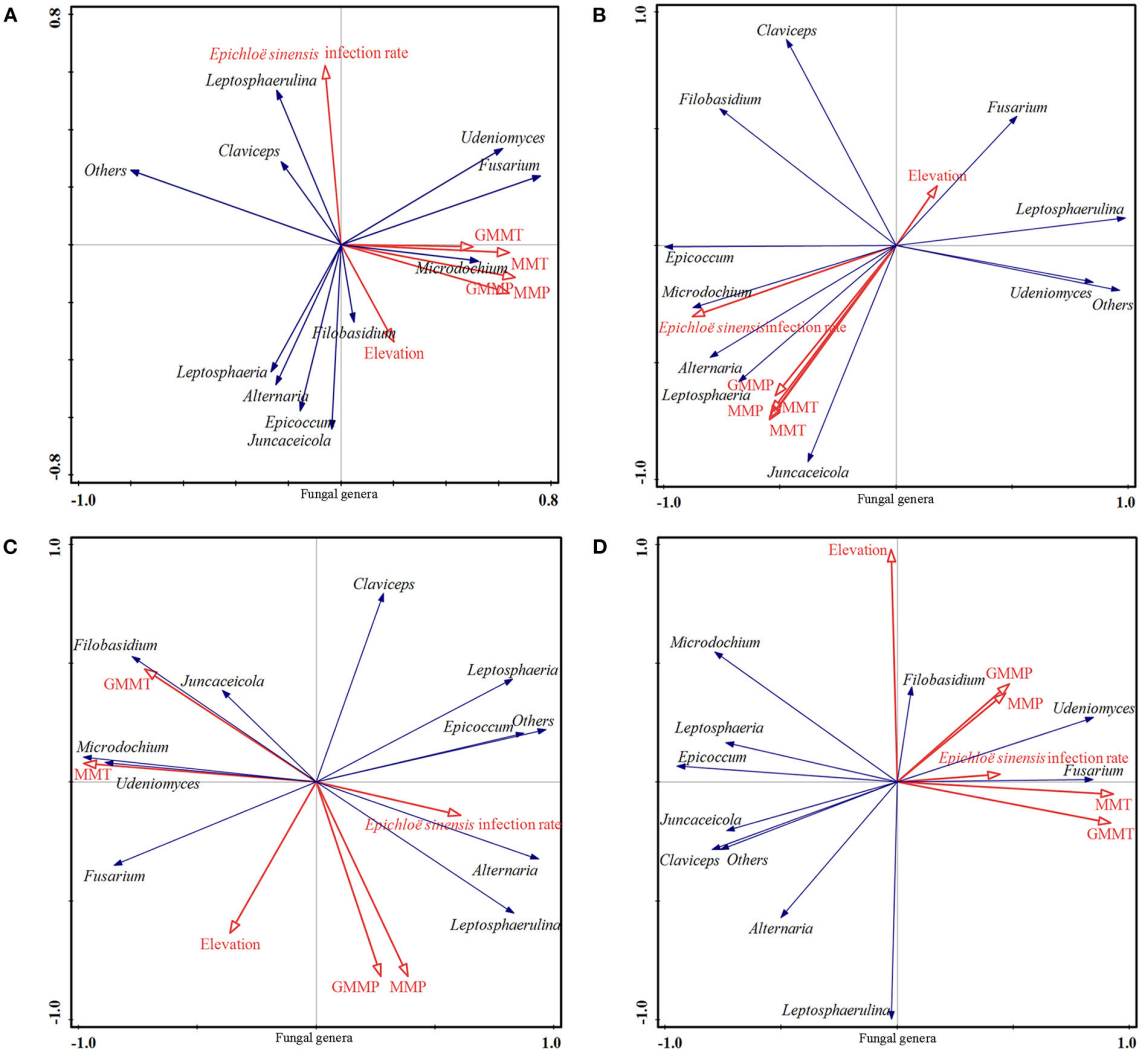
Effect of environmental factors on fungal genera. **(A)** on overall elevation (2,589–5,197 m). **(B)** on high elevation (2,589–3,000 m); **(C)** on medium-high elevation (3,000–4,500 m); **(D)** and on extremely high elevation (4,500–5,197 m). The microbial parameters (expressed as response variables in the RDA) were presented as black line vectors, and the environmental factors (explanatory variables) were presented as red line vectors. MMT, monthly mean temperature; MMP, monthly mean precipitation; GMMT, growing monthly mean temperature; GMMP, growing monthly mean precipitation.

Redundancy analysis, as tested by Monte Carlo permutation on the bacterial phyla (Figure 5), fungal phyla (Figure 6), bacterial genera (Figure 7), and fungal genera (Figure 8) of samples at the overall elevation (2,589–5,197 m), high elevation (2,589–3,000 m), medium-high elevation (3,000–4,500 m), and extremely high elevation (4,500–5,197 m), identified the environmental factors (MMT, GMMT, MMP, and GMMP) that were significantly correlated with the microbial community. Environmental factors (GMMP, MMP, GMMT, and MMT) were positively correlated with the abundances of Chloroflexi, Elusimicrobiota, Acidobacteriota, Verrucomicrobiota, and Bateroidota at overall elevation (Figure 5A), high elevation (Figure 5B), and extremely high elevation (Figure 5D), however, negatively correlated with the abundance of Cyanobacteria at overall elevation, high elevation, and extremely high elevation (Figures 5A,B,D). The elevation was positively correlated with the abundances of Cyanobacteria at overall elevation and medium-high elevation (Figures 5A,C), whereas negatively correlated with the abundances of Cyanobacteria at high elevation and extremely high elevation (Figures 5B,D). At medium-high elevation group, GMMT and MMT were positively correlated with the abundance of Elusimicrobiota, Acidobacteriota, Chloroflexi, and Actinobacteriota; however, GMMP and MMP were negatively correlated with them (Figure 5C).

Environmental factors (MMT, GMMT, MMP, and GMMP) were significantly correlated with the fungal community at the phyla levels (Figure 6). Environmental factors (GMMP, MMP, GMMT, and MMT) were positively correlated with the abundances of Ascomycota (Figures 6A,B,D). The elevation was positively correlated with the abundance of Ascomycota, Basidiomycota, whereas *E. sinensis* infection rate was negatively correlated with the abundance of Ascomycota and Basidiomycota (Figures 6A,D). At the medium-high elevation, the GMMP and MMP were positively correlated with the abundance of Ascomycota, Chytridiomycota, and Mortierellomycota, whereas negatively correlated with the abundances of Basidiomycota and Glomeromycota (Figure 6C), GMMT and MMT were positively correlated with the abundances of Basidiomycota and Glomeromycota, whereas negatively correlated with the abundances of Ascomycota, Chytridiomycota, and Mortierellomycota (Figure 6C).

As shown in Figure 7, environmental factors (GMMP, MMP, GMMT, and MMT) were positively correlated with the abundances of *Amaricoccus, Sphingomonas, Pseudomonas, Paracoccus, Pedobacter, Massilia, Duganella, and Hymenobacter*, whereas negatively correlated with the abundances of *unidentified_Chloroplast* and *unidentified_Mitochondria* at overall elevation, high elevation, and extremely high elevation (Figures 7A,B,D). For all four groups, the elevation was positively correlated with the abundances of *Pseudomonas* and *Duganella*; however, *E. sinensis* infection rate was negatively correlated with them (Figure 7). At medium-high elevation group, GMMT and MMT were positively correlated with the abundance of *Pedobacter, Massilia, Amaricoccus, and Paracoccus*, whereas GMMP and MMP were negatively correlated with them (Figure 7C). The GMMP and MMP were positively correlated with the abundance of *Duganella, Pseudomonas*, and *Sphingomonas*; however, GMMP and MMP were negatively correlated with them (Figure 7C).

As shown in Figure 8, environmental factors (GMMP, MMP, GMMT, and MMT) were positively correlated with the abundances of *Juncaceicola, Filobasidium, Microdochium, Fusarium*, and *Udeniomyces* at overall elevations, high elevations, and extremely high elevation (Figures 8A,B,D). For all four groups, elevation was positively correlated with the abundances of *Fusarium* and *Udeniomyces*, and *E. sinensis* infection rate was negatively correlated with the abundances of *Fusarium, Udeniomyces* (Figure 8). At the medium-high elevation, GMMP and MMP were positively correlated with the abundance of *Leptosphaerulina, Alternaria, Epicoccum*, and *Leptosphaeria*, whereas negatively correlated with the abundances of *Juncaceicola, Filobasidium, Microdochium*, and *Udeniomyces*. GMMP and MMP were positively correlated with the abundance of *Juncaceicola, Filobasidium, Microdochium*, and *Udeniomyces*, whereas negatively correlated with the abundances of *Leptosphaerulina, Alternaria, Epicoccum*, and *Leptosphaeria* (Figure 8C).

## Discussion

There is a rich microbial community comprising a diverse range of bacteria (Truyens et al., 2015) and fungi (Shearin et al., 2018) on the surfaces and interiors of plant seeds. In this study, we characterized the seed microbiota from 14 different locations on the Qinghai-Tibet Plateau. A total of 64 bacterial phyla and six fungal phyla were found in the *F. sinensis* seeds by high-throughput sequencing. Moreover, α-diversity identified the important environment factors (temperature and precipitation) and β-diversity further showed that the microbial community structure varied greatly among different collection sites of the Qinghai-Tibet Plateau, which demonstrated that the microbiota diversity of *F. sinensis* seeds had a strong relationship with the geographical distribution. In general, the results suggested that the microbiome in different *F. sinensis* seed lots had different components. Previous research found a total of 54 genera and 129 species of bacteria were isolated from the surface and interior of more than 30 types of crop seeds. Among these bacteria, Proteobacteria was the main phylum, and Firmicutes, Actinobacteria, and Bacteroidetes were the second most common (Liu et al., 2012). Our study was consistent with the results of previous studies. Proteobacteria play a key role in phylogenetic, ecological, and pathogenic processes and participate in energy metabolism, including oxidation and photosynthesis of organic and inorganic compounds (Zhang et al., 2018). Cyanobacteria produce phycoerythrin and phycocyanin, two photosynthetic pigments characteristic of Rhodophyta or red algae, and one or two other smaller groups (Edelman et al., 1967). Cyanobacteria are also well recognized as producers of a wide array of bioactive metabolites including toxins and potential drug candidates (Walton and Berry, 2016). Thus, Cyanobacteria may improve plant growth and seed germination (Chua et al., 2019). Bacteroidetes are increasingly regarded as specialists in the degradation of high molecular weight organic matter, namely, proteins and carbohydrates (Thomas et al., 2011). A possible explanation for our findings is that the climate of the Qinghai-Tibet Plateau leads to more Cyanobacteria in the *F. sinensis* seeds. The higher amount of Cyanobacteria is more conducive to photosynthesis in *F. sinensis*, which makes it better adapted for growth in the Qinghai-Tibet Plateau. Ascomycota and Basidiomycota were the most abundant fungi, consistent with the previous studies conducted on alpine meadows in the Yushu Tibetan Autonomous Prefecture, which showed that most fungi belonged to Ascomycota (Chen et al., 2017), Ascomycota mainly decompose cellulose and lignin, and its growth may depend on more readily available energy sources, such as soluble carbohydrates (Osono and Takeda, 2002). Basidiomycota mainly produce lignin-modifying enzymes that degrade lignin (Osono et al., 2003). Ascomycota and Basidiomycota are widely distributed in plants, aquatic ecosystems, and soil, in different proportions (Vandenkoornhuyse et al., 2002). These microorganisms play the important roles in seed health and production.

The coexistence of microbial symbionts and hosts contributes to host adaptation to the natural environment. In turn, the composition of the plant microbial community responds to the environment and the host, making it possible for the plant to benefit. Seeds are involved in the transmission of microorganisms from one plant generation to another and consequently may act as the initial inoculum source for the plant microbiota (Rezki et al., 2018). Seeds have evolved in association with diverse microbial assemblages that may influence plant growth and health (Gibbons et al., 2013). Microbial communities and structures vary and can be influenced by many factors. Previous studies have found that both seed and soil types affect microbial communities (Buyer et al., 1999). Different environmental conditions affect the propagation, infection, and transmission of various microbes, and different species carry different types and numbers of microbes. The same seed can carry different fungi under different growth conditions (Thomas et al., 2016). Environmental factors have different effects on the abundance and diversity of seed microorganisms. High-altitude ecosystems are generally characterized by low temperatures, variable precipitation, decreased atmospheric pressure, and soil nutrient stress, which have major impacts on biodiversity (Moran-Tejeda et al., 2013). We found that precipitation and temperature were the dominant drivers of the bacterial and fungal diversity gradients (Nottingham et al., 2018; Shen et al., 2020). Seeds of different ecotypes carried abundant amounts of different microorganisms that adapt to different environments, including elevation, temperature, and humidity. Some studies found that microbial species richness and diversity were closely correlated with temperature and that microbial diversity decreased under extreme and polar temperatures (Sharp et al., 2014). In our study, temperature and precipitation were the main environmental factors that drive variations in the microbial community. Further correlation analysis showed that the most abundant bacterial phyla were significantly correlated with temperature and the most abundant fungal phyla were significantly correlated with precipitation.

A possible explanation is that temperature and precipitation became the dominant environmental factors that affect bacterial and fungal community diversity in the Qinghai-Tibet Plateau, which is characterized by low temperature and low precipitation due to high elevation. Precipitation has a strong impact on humidity, temperature, and other conditions in the air, which influence the growth of microorganisms. Some studies have shown that certain bacterial populations respond immediately to an increase in soil moisture once the monsoon rains arrive, although the relative abundances of most bacterial phyla showed less variation during the course of the study (McHugh et al., 2014). In this study, it is possible that precipitation affected local atmospheric conditions such as temperature and humidity, which further affected the abundance and diversity of the microbial communities. The *E. sinensis* infection rate in *F. sinensis* seeds on the Qinghai-Tibet Plateau was systematically investigated. The asexual *Epichloë* is extant in the embryos of seeds and is transmitted through the seeds (Wang et al., 2018). There have been extensive studies and reports on the influence of *Epichloë* endophytes on host performance, including disease and insect resistance, and cold and drought tolerance (Chen et al., 2016; Xia et al., 2018; Bu et al., 2019). However, there are relatively few reports on the effects of *Epichloë* endophytes on microbial communities, and research results are inconsistent. One study suggested that *Epichloë* endophytes can affect the microbial diversity in the roots and rhizosphere (Liu et al., 2021). Another study has reported that *E. festucae* var. *lolii* strains AR1 and AR37 significantly changed the rhizosphere bacterial community composition of the host perennial ryegrass but had no significant effect on the Pseudomonas community (Li et al., 2017). These studies have shown that *Epichloë* endophytes had different effects on different microbial communities. However, in this study, *E. sinensis* infection rate did not affect the relative abundance of the most abundant microbiota. In this study, *F. sinensis* seeds were obtained from a high-altitude areas of the Qinghai-Tibet Plateau, where the temperature and precipitation differ from those of the plain area. Correlation analysis showed that the *Epichloë sinensis* infection rate was significantly correlated with temperature and elevation, which further confirmed our hypothesis. Thus, this study provides some clues for studying the effects of *Epichloë* endophytes on seed microbiota.

The *E. sinensis* infection rate was also affected by environmental factors, which were significantly negatively correlated with elevation and positively correlated with temperature. Bacon and Siegel (1988) noted a decrease in the seed endophyte infection rate and vegetative tissue of tall fescue after the plants had experienced hot and dry summers and cold winters. Ju et al. found through growth chamber experiments that temperature appears to be a major variable that affects the fluctuation of endophyte frequency in plant tissues (Ju et al., 2006). Previous studies found that high concentrations of carbon dioxide significantly affect the infection frequency of endophytic fungi in tall fescue, and precipitation may promote this grass–fungal symbiosis, leading to higher endophyte infection frequency (Brosi et al., 2011). These studies indicate that endophytic fungi are susceptible to various environmental factors. In this study, *F. sinensis* seed lots were obtained from different sites on the Qinghai-Tibet Plateau; possibly, the combination of both host genotype and environment interactions led to the variations among the microbiomes of these 14 seed lots. The relationship between endophyte infection rate and host growth environment has not been confirmed as the research has been limited. Some reports have shown that endophyte infection rates of *Elymus tangutorum, Lolium rigidum*, and *F. rubra* decreased with increasing elevation (Bazely et al., 2007; Kirkby et al., 2011; Shi et al., 2020). However, other studies on *F. ovina, F. eskia, Deschampsia flexuosa*, and *Poa trivialis* did not find a relationship between endophyte infection rate and elevation (Bazely et al., 2007; Granath et al., 2007; Gonzalo-Turpin et al., 2010). Some studies have shown that the frequency of *E. alsodes* in *Poa alsodes* was positively correlated with July maximum temperatures, July precipitation, and soil nitrogen and phosphorous (Shymanovich and Faeth, 2019).

## Conclusion

The microbiota of *F. sinensis* seeds of 14 different ecotypes collected from the Qinghai-Tibet Plateau were rich in diversity and were significantly affected by two environmental factors: temperature and precipitation; however, at the medium-high elevation (3,000–4,500 m), the impact of temperature and precipitation on microbial community is different. *E. sinensis* infection rates in the host *F. sinensis* seeds varied among different geographic locations, which varied in environmental factors affecting host growth, including elevation and temperature. However, the specific mechanisms of the variations require further investigation.

## Data availability statement

The data presented in the study are deposited in the NCBI Sequence Read Archive (SRA) repository, accession number BioProject ID PRJNA844205.

## Author contributions

PT designed the experiment, revised, and polished the manuscript and provide guidance for data analysis. YG conducted data analysis and draft the manuscript. YC and QZ provided the experimental materials. ZN provided guidance for samples collection and experimental design. YL and JL helped in data analysis. All authors contributed to the article and approved the submitted version.

## Funding

This study was supported by the National Nature Science Foundation of China (31971768), Gansu transportation department science and technology project 2021-07 and Gansu transportation department science and technology project 2021-28.

## Conflict of interest

The authors declare that the research was conducted in the absence of any commercial or financial relationships that could be construed as a potential conflict of interest.

## Publisher's note

All claims expressed in this article are solely those of the authors and do not necessarily represent those of their affiliated organizations, or those of the publisher, the editors and the reviewers. Any product that may be evaluated in this article, or claim that may be made by its manufacturer, is not guaranteed or endorsed by the publisher.
