# Supplementary material for: The microbiota diversity of Festuca sinensis seeds in Qinghai-Tibet Plateau and their relationship with environments
Source: Front Microbiol. 2022 Aug 3;13:956489. doi: 10.3389/fmicb.2022.956489 (PMC9382023; doi:10.3389/fmicb.2022.956489)
Supplement: Supplementary file 1 [file Data_Sheet_1.docx]

Supplementary Material

**Supplementary Excel.** Meteorological data (MMT, monthly mean temperature, MMP, monthly mean precipitation, GMMT, growing monthly mean temperature, GMMP, growing monthly mean precipitation.**)**

# Supplementary Figures and Tables


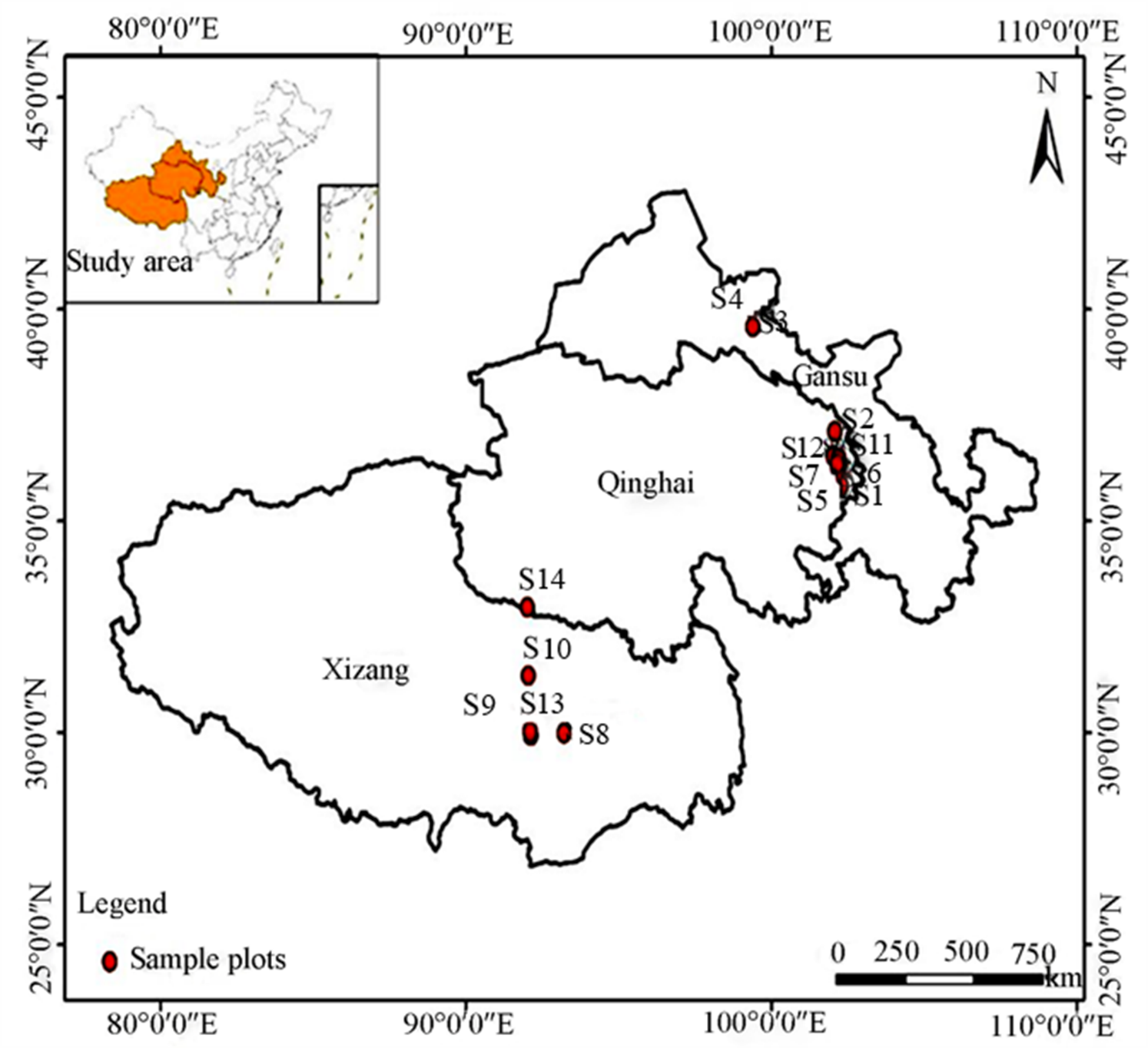
 Supplementary Figure S1. Approximate geographical location of collected the each Festuca sinensis seeds from different ecotypes.

Note: the red solid circle on the map stand for sample collection location

#
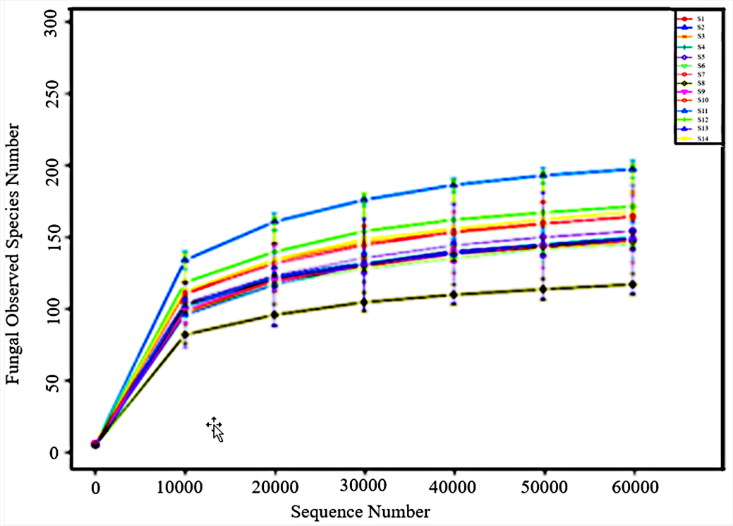


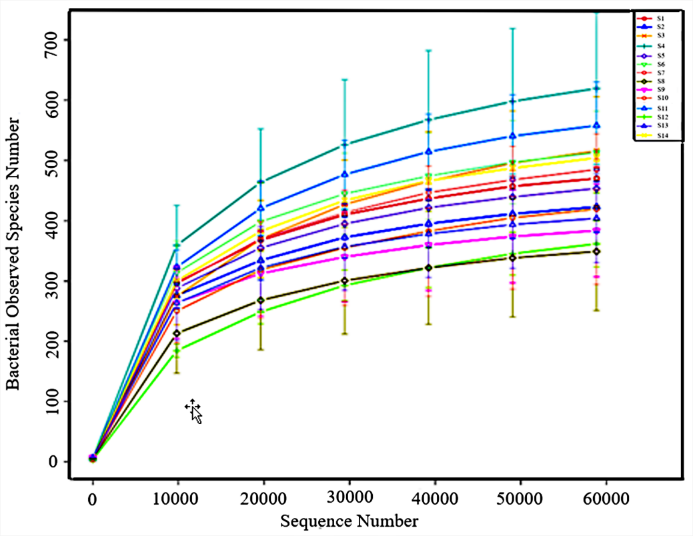


**(A)**

**(B)**

Supplementary Figure S2: Rarefaction (A) and (B) the depth of sequencing.

## Supplementary Tables

**Supplementary Table S1** Coverage estimator bacteria and fungi communities in seeds

| Ecotypes | Coverage estimator of bacteria | Coverage estimator of fungi |
| --- | --- | --- |
| S1 | 0.999 | 1 |
| S2 | 0.999 | 1 |
| S3 | 0.998 | 1 |
| S4 | 0.998 | 1 |
| S5 | 0.999 | 1 |
| S6 | 0.999 | 1 |
| S7 | 0.999 | 1 |
| S8 | 0.999 | 1 |
| S9 | 0.999 | 1 |
| S10 | 0.999 | 1 |
| S11 | 0.999 | 1 |
| S12 | 0.999 | 1 |
| S13 | 0.999 | 1 |
| S14 | 0.998 | 1 |

**Supplementary Table S2**. The relative abundance of the most abundant bacteria and fungi phyla

| Ecotypes | Bacterial phyla% | | |  | Fungal phyla% | |
| --- | --- | --- | --- | --- | --- | --- |
|  | **Proteobacteria** | **Cyanobacteria** | **Bacteroidota** |  | **Ascomyota** | **Basidiomycota** |
| S1 | 48 ± 0.03cd | 38 ± 0.09ab | 7 ± 0.03bc |  | 46 ± 0.07abc | 17 ± 0.04ab |
| S2 | 66 ± 0.08abc | 16 ± 0.12bcd | 11 ± 0.04b |  | 47 ± 0.01abc | 12 ± 0.01abc |
| S3 | 50 ± 0.02cd | 46 ± 0.02a | 1 ± 0.00bc |  | 33 ± 0.04c | 9 ± 0.01bc |
| S4 | 48 ± 0.01cd | 38 ± 0.01ab | 8 ± 0.00bc |  | 48 ± 0.11abc | 20 ± 0.04a |
| S5 | 40 ± 0.06d | 30 ± 0.15abc | 7 ± 0.02bc |  | 53 ± 0.01abc | 14 ± 0.01abc |
| S6 | 64a ± 0.08bc | 20 ± 0.13abcd | 8 ± 0.03bc |  | 49 ± 0.05abc | 12 ± 0.01abc |
| S7 | 60 ± 0.14bc | 28 ± 0.14abc | 7 ± 0.01bc |  | 65 ± 0.04ab | 14 ± 0.02abc |
| S8 | 67 ± 0.09abc | 19 ± 0.08abcd | 4 ± 0.01bc |  | 51 ± 0.06abc | 6 ± 0.00c |
| S9 | 74 ± 0.02ab | 1 ± 0.00d | 19 ± 0.02a |  | 67 ± 0.05a | 13 ± 0.03abc |
| S10 | 59 ± 0.04bcd | 29 ± 0.04abc | 4 ± 0.01bc |  | 59 ± 0.08abc | 14 ± 0.03abc |
| S11 | 53 ± 0.02cd | 43 ± 0.02ab | 3 ± 0.00bc |  | 44 ± 0.04abc | 11 ± 0.01abc |
| S12 | 55 ± 0.02bcd | 43 ± 0.02ab | 1 ± 0.00c |  | 36 ± 0.04bc | 17 ± 0.03ab |
| S13 | 82 ± 0.03a | 4 ± 0.01cd | 7 ± 0.01bc |  | 61 ± 0.09abc | 17 ± 0.06abc |
| S14 | 56 ± 0.04bcd | 29 ± 0.06abc | 7 ± 0.03bc |  | 50 ± 0.03abc | 19 ± 0.01a |

Note: The values in the table are relative abundances, and different lower case letters indicate significance among different ecotype (P<0.05)

**Supplementary Table S3.** KMO and barllett's test for PCA

|  | | bacteria |  | fungi |
| --- | --- | --- | --- | --- |
| Kaiser-Mayer-Olkin Measure of Sampling Adequacy | | 0.847 |  | 0.938 |
| Bartlett’s sphericity test | Approx. Chi-Square | 135753.448 |  | 118534.075 |
|  | Df | 55 |  | 861 |
|  | Sig | 0.000 |  | 0.000 |

**Supplementary Table S4**. Redundancy analysis of the correlation between environmental factors and bacterial phyla at different elevations

|  |  | Axis | Eigenvalues | Pseudo-canonical correlation | Explained variation (cumulative) % | Explained fitted variation (cumulative) % |
| --- | --- | --- | --- | --- | --- | --- |
| Bacterial phyla | Overall elevation (2589-5197) | 1 | 0.7083 | 0.9017 | 70.83 | 92.84 |
|  |  | 2 | 0.0322 | 0.6259 | 74.05 | 97.06 |
|  |  | 3 | 0.0131 | 0.7193 | 75.35 | 98.77 |
|  |  | 4 | 0.0085 | 0.6701 | 76.2 | 99.89 |
|  | High elevation (2589-3000) | 1 | 0.9025 | 1 | 90.25 | × |
|  |  | 2 | 0.0747 | 1 | 97.72 |  |
|  |  | 3 | 0.0132 | 1 | 99.03 |  |
|  |  | 4 | 0.0097 | 1 | 100.00 |  |
|  | Medium-high elevation (3000-4500) | 1 | 0.8382 | 1 | 83.82 | × |
|  |  | 2 | 0.1180 | 1 | 95.62 |  |
|  |  | 3 | 0.0438 | 1 | 100.00 |  |
|  |  | 4 | × | × | × |  |
|  | Extremely high elevation (4500-5197) | 1 | 0.6069 | 1 | 60.69 | × |
|  |  | 2 | 0.1907 | 1 | 79.76 |  |
|  |  | 3 | 0.1767 | 1 | 97.44 |  |
|  |  | 4 | 0.0256 | 1 | 100.00 |  |

**Supplementary Table S5**. Redundancy analysis of the correlation between environmental factors and fungal phyla at different elevations

|  |  | Axis | Eigenvalues | Pseudo-canonical correlation | Explained variation (cumulative) % | Explained fitted variation (cumulative) % |
| --- | --- | --- | --- | --- | --- | --- |
| Fungal phyla | Overall elevation (2589-5197) | 1 | 0.6147 | 0.8319 | 61.47 | 89.8 |
|  |  | 2 | 0.0693 | 0.7933 | 68.41 | 99.92 |
|  |  | 3 | 0.0005 | 0.5797 | 68.46 | 100 |
|  |  | 4 | 0 | 0.3052 | 68.46 | 100 |
|  | High elevation (2589-3000) | 1 | 0.9864 | 1 | 98.64 | × |
|  |  | 2 | 0.0117 | 1 | 99.82 |  |
|  |  | 3 | 0.0017 | 1 | 99.99 |  |
|  |  | 4 | 0.0001 | 1 | 100.00 |  |
|  | Medium-high elevation (3000-4500) | 1 | 0.8629 | 1 | 86.29 | × |
|  |  | 2 | 0.1210 | 1 | 98.38 |  |
|  |  | 3 | 0.0162 | 1 | 100.00 |  |
|  |  | 4 | × | × | × |  |
|  | Extremely high elevation (4500-5197) | 1 | 0.8345 | 1 | 83.45 | × |
|  |  | 2 | 0.1319 | 1 | 96.63 |  |
|  |  | 3 | 0.0324 | 1 | 99.87 |  |
|  |  | 4 | 0.0013 | 1 | 100.00 |  |

**Supplementary Table S6**. Redundancy analysis of the correlation between environmental factors and bacterial genera at different elevations

|  |  | Axis | Eigenvalues | Pseudo-canonical correlation | Explained variation (cumulative) % | Explained fitted variation (cumulative) % |
| --- | --- | --- | --- | --- | --- | --- |
| Bacterial genera | Overall elevation (2589-5197) | 1 | 0.7501 | 0.9238 | 75.01 | 93.98 |
|  |  | 2 | 0.0304 | 0.7152 | 78.05 | 97.78 |
|  |  | 3 | 0.0142 | 0.6337 | 79.47 | 99.56 |
|  |  | 4 | 0.0025 | 0.7179 | 79.73 | 99.88 |
|  | High elevation (2589-3000) | 1 | 0.6362 | 1 | 63.62 | × |
|  |  | 2 | 0.2640 | 1 | 90.02 |  |
|  |  | 3 | 0.0812 | 1 | 98.14 |  |
|  |  | 4 | 0.0186 | 1 | 100.00 |  |
|  | Medium-high elevation (3000-4500) | 1 | 0.6678 | 1 | 66.78 | × |
|  |  | 2 | 0.2566 | 1 | 92.44 |  |
|  |  | 3 | 0.0756 | 1 | 100.00 |  |
|  |  | 4 | × | × | × |  |
|  | Extremely high elevation (4500-5197) | 1 | 0.7978 | 1 | 79.78 | × |
|  |  | 2 | 0.1740 | 1 | 97.18 |  |
|  |  | 3 | 0.0241 | 1 | 99.59 |  |
|  |  | 4 | 0.0041 | 1 | 100.00 |  |

**Supplementary Table S7**. Redundancy analysis of the correlation between environmental factors and fungal genera at different elevations

|  |  | Axis | Eigenvalues | Pseudo-canonical correlation | Explained variation (cumulative) % | Explained fitted variation (cumulative) % |
| --- | --- | --- | --- | --- | --- | --- |
| Fungal genera | Overall elevation (2589-5197) | 1 | 0.4834 | 0.8365 | 48.34 | 78.37 |
|  |  | 2 | 0.0917 | 0.7077 | 57.51 | 93.23 |
|  |  | 3 | 0.0283 | 0.8487 | 60.34 | 97.82 |
|  |  | 4 | 0.0097 | 0.4865 | 61.32 | 99.4 |
|  | High elevation (2589-3000) | 1 | 0.7228 | 1 | 72.28 | × |
|  |  | 2 | 0.2357 | 1 | 95.85 |  |
|  |  | 3 | 0.0365 | 1 | 99.50 |  |
|  |  | 4 | 0.0050 | 1 | 100.00 |  |
|  | Medium-high elevation (3000-4500) | 1 | 0.7334 | 1 | 73.34 | × |
|  |  | 2 | 0.1766 | 1 | 91.00 |  |
|  |  | 3 | 0.0900 | 1 | 100.00 |  |
|  |  | 4 | × | × | × |  |
|  | Extremely high elevation ( 4500-5197) | 1 | 0.4949 | 1 | 49.49 | × |
|  |  | 2 | 0.2938 | 1 | 78.87 |  |
|  |  | 3 | 0.1729 | 1 | 96.16 |  |
|  |  | 4 | 0.0384 | 1 | 100.00 |  |

**Table S8.** Importance ranking and significance test of environmental factor explanation on the composition of microbial communities.

|  | | |  | Environmental factors | [Importance](javascript:;) [ranking](javascript:;) | Explains % | F | P |
| --- | --- | --- | --- | --- | --- | --- | --- | --- |
| Overall elevation (2589-5197) | Bacterial phyla | | GMMP | 1 | 42.4 | 8.8 | **0.002** |  |
|  |  |  | GMMT | 2 | 21.0 | 7.4 | **0.012** |  |
|  |  |  | MMT | 3 | 8.3 | 1.8 | 0.192 |  |
|  |  |  | MMP | 4 | 2.4 | 0.8 | 0.468 |  |
|  |  |  | *Epichloë sinensis* infection rate | 5 | 1.3 | 0.4 | 0.712 |  |
|  |  |  | Elevation | 6 | 1.0 | 0.3 | 0.822 |  |
|  | Fungal phyla | | MMP | 1 | 29.3 | 5.0 | **0.03** |  |
|  |  |  | MMT | 2 | 23.2 | 5.8 | **0.028** |  |
|  |  |  | *Epichloë sinensis* infection rate | 3 | 7.4 | 1.3 | 0.248 |  |
|  |  |  | GMMP | 4 | 3.2 | 0.8 | 0.458 |  |
|  |  |  | Elevation | 5 | 2.8 | 0.6 | 0.492 |  |
|  |  |  | GMMT | 6 | 2.5 | 0.6 | 0.552 |  |
|  | Bacterial genera | | MMP | 1 | 48.4 | 11.3 | **0.002** |  |
|  |  |  | GMMT | 2 | 17.9 | 7.7 | **0.012** |  |
|  |  |  | MMT | 3 | 10.4 | 2.8 | 0.108 |  |
|  |  |  | *Epichloë sinensis* infection rate | 4 | 2.1 | 0.9 | 0.406 |  |
|  |  |  | GMMP | 5 | 0.7 | 0.4 | 0.712 |  |
|  |  |  | Elevation | 6 | 0.4 | 0.1 | 0.966 |  |
|  | Fungal genera | | GMMP | 1 | 21.7 | 3.3 | **0.036** |  |
|  |  |  | MMP | 2 | 17.8 | 3.4 | 0.05 |  |
|  |  |  | MMT | 3 | 7.5 | 1.2 | 0.324 |  |
|  |  |  | *Epichloë sinensis* infection rate | 4 | 9.2 | 1.9 | 0.158 |  |
|  |  |  | Elevation | 5 | 3.3 | 0.6 | 0.656 |  |
|  |  |  | GMMT | 6 | 2.1 | 0.4 | 0.772 |  |
|  |  | | **On all axes** | | | **F** | **P** |  |
| High elevation (2589-3000) | Bacterial phyla | | × | | | <0.1 | 1 |  |
|  | Fungal phyla | |  |  |  | <0.1 | 1 |  |
|  | Bacterial genera | |  |  |  | <0.1 | 1 |  |
|  | Fungal genera | |  |  |  | <0.1 | 1 |  |
| Medium-high elevation (3000-4500) | Bacterial phyla | | × | | | <0.1 | 1 |  |
|  | Fungal phyla | |  |  |  | <0.1 | 1 |  |
|  | Bacterial genera | |  |  |  | <0.1 | 1 |  |
|  | Fungal genera | |  |  |  | <0.1 | 1 |  |
| Extremely high elevation (4500-5197) | Bacterial phyla | | × | | | <0.1 | 1 |  |
|  | Fungal phyla | |  |  |  | <0.1 | 1 |  |
|  | Bacterial genera | |  |  |  | <0.1 | 1 |  |
|  | Fungal genera | |  |  |  | <0.1 | 1 |  |

Note: MMT, monthly mean temperature, MMP, monthly mean precipitation, GMMT, the growing monthly mean temperature and GMMP, growing monthly mean precipitation.
